# Identification of mRNA Prognostic Markers for TGCT by Integration of Co-Expression and CeRNA Network

**DOI:** 10.3389/fendo.2021.743155

**Published:** 2021-09-21

**Authors:** Fang Zhu, Zhizhong Liu, Qianyin Zhou, Jingyu Fan, Dai Zhou, Liu Xing, Hao Bo, Le Tang, Liqing Fan

**Affiliations:** ^1^National Health Commission Key Laboratory of Human Stem Cell and Reproductive Engineering, Institute of Reproductive and Stem Cell Engineering, Central South University, Changsha, China; ^2^Hunan Cancer Hospital, Department of Urology, The Affiliated Cancer Hospital of Xiangya School of Medicine of Central South University, Changsha, China; ^3^Department of Chemistry and Biochemistry, University of South Carolina, Columbia, SC, United States; ^4^Clinical Research Center for Reproduction and Genetics in Hunan Province, Reproductive and Genetic Hospital of China International Trust and Investment Corporation (CITIC) Xiangya, Changsha, China; ^5^Reproductive Medicine Center, Maternal and Child Health Care Hospital of Hunan Province, Changsha, China

**Keywords:** testicular germ cell tumors, prognostic markers, risk factor, immune cell infiltration, chemotherapy resistance, competitive endogenous RNAs network

## Abstract

Testicular germ cell tumor (TGCT) is the most common malignant tumor in young men and is associated with poor prognosis. We assessed the RNA expression profiles of 13 TGCT tissues and 4 adjacent normal tissues by transcriptome sequencing to identify novel prognostic biomarkers. We detected several differentially expressed mRNAs in TGCT that were functionally annotated by GO and KEGG enrichment analyses to tumorigenesis-related processes such as immunity and chemotherapeutic resistance. An mRNA-lncRNA-miRNA regulatory network was constructed using RNA-Seq data and public databases, and integrated with TCGA database to develop a prediction model for metastasis and recurrence. Finally, GRK4, PCYT2 and RGSL1 were identified as predictive markers of survival and therapeutic response. In conclusion, we found several potential predictors for TGCT prognosis and immunotherapeutic response by ceRNA network analysis.

## Introduction

Testicular germ cell tumors (TGCTs) are prevalent among males between the ages of 20 and 35 years, and the incidence rates are increasing globally. Approximately 90-95% of testicular cancers are TGCTs that are derived from the germ cell lineage (1). Histologically, TGCTs are classified into the seminomas (SEM) and non-seminomas (non-SEM), of which the latter can be sub-divided into teratomas, yolk sac tumors, choriocarcinomas, embryonal carcinomas (ECs) and mixed tumors (2). Non-SEM has a high risk of metastasis and recurrence, and the ECs in particular that originate from the malignant testicular stem cells are the most aggressive TGCT subtype due to vascular invasion (3–5). However, the mechanisms underlying the poor prognosis of TGCTs are poorly understood.

Although TGCT is generally sensitive to cisplatin-based chemotherapy, approximately 20% of the patients are unresponsive to the treatment and have significantly worse prognoses or a high risk of recurrence, along with complications such as peripheral neuropathy, hormonal disturbances, sexual dysfunction, infertility, cognitive impairment and psychosocial effects (6–9). Despite the familial nature of TGCTs, the driver genes for tumorigenesis or metastasis have not been identified so far that may be potentially targeted for therapy (10). Currently, most TGCT-related deaths can be attributed to metastasis and cisplatin resistance (11). Therefore, it is crucial to identify novel biomarkers of TGCTs to improve early diagnosis and relapse-free survival (RFS).

Recent studies have shown that in addition to mRNAs, microRNAs (miRNAs) and long non-coding RNAs (lncRNAs) are involved in the pathogenesis of TGCTs (12, 13). Competing endogenous RNA (ceRNA) networks play a vital role in the regulation of oncogenic pathways (14). In this study, we identified multiple dysregulated RNAs in TGCTs relative to adjacent normal tissues by transcriptome sequencing. The differentially expressed mRNAs (DEmRNAs) were associated with immune cell infiltration during TGCTs progression. We constructed a ceRNA network for TGCTs based on these dysregulated mRNAs and lncRNAs, and a model for predicting RFS and immunotherapeutic responses by integrating the ceRNAs network and clinical data from public databases. These findings provide new insights into the molecular basis of TGCTs pathogenesis.

## Materials and Methods

### Clinical Specimens

Thirteen TGCT tissue samples (11 from SEM and 2 from non-SEM patients with predominantly malignant embryonal carcinoma) and 4 para-carcinoma tissues were obtained from the Department of Urology, the Affiliated Cancer Hospital of Xiangya School of Medicine of Central South University (**Table 1**). Fresh tissues were collected and snap frozen in liquid nitrogen. The study was approved by the Ethics Committee, and informed consent was obtained from each patient.

**Table 1:** Clinical case information of 17 testicular tissues in our sequencing.

Sample name	Age	Left /right	Pathological type	Ki67 positive rate	The broken end of spermatic cord is involved or not
SEM-3	35	Left	Seminoma	70%	Not involved
SEM-4	44	Right	Seminoma	60%	Not involved
SEM-5	28	Right	Seminoma	60%	Not involved
SEM-6	27	Right	Seminoma	60%	Not involved
SEM-8	32	Left	Seminoma	40%	Not involved
SEM-2	58	Left	Seminoma	–	Not involved
SEM-9	33	Right	Seminoma	–	Not involved
SEM-10	31	Right	Seminoma	80%	Not involved
SEM-12	44	Right	Seminoma	80%	Not involved
SEM-13	42	Right	Seminoma	60%	Not involved
SEM-14	52	Left	Seminoma	35%	Not involved
Non-SEM-2	45	Left	Embryonic tumor	–	Not involved
Non-SEM-3	47	Left	Embryonic tumor	80%	Not involved
Non-tumor-1	44	Right	para-carcinoma tissue	–	–
Non-tumor -2	45	Left	para-carcinoma tissue	–	–
Non-tumor -3	28	Right	para-carcinoma tissue	–	–
Non-tumor -4	32	Left	para-carcinoma tissue	–	–

### RNA-Sequencing

Total RNA was extracted from tissue samples using Trizol reagent (Thermo, USA), and 1µL per sample was used to determine the concentration using a NanoDrop 1000 spectrophotometer. After removing rRNAs (Ribo-Zero Gold rRNA Removal Kit, MRZG12324, Illumina), the remaining mRNAs and ncRNAs were purified using Agencourt RNA Clean XP Beads. The enriched mRNA and ncRNA fragments were cut into shorter sequences using a fragmentation buffer and reverse-transcribed using the Transcriptor First-strand cDNA synthesis Kit (Roche, USA). The cDNA fragments were purified to repair the ends add poly (A) and ligate the Illumina sequencing joints (QiaQuick PCR extraction kit). The second-strand cDNA was digested with uracil-N-glucanase, and amplified by PCR. The Sensitivity DNA assay Kit (Agilent Technologies) was used to analyze the quality of the library. Finally, ABI StepOnePlus Real-Time PCR System (Life Technologies) was used to quantify and pool the sequences, and the DNA sequenced on the Illumina HiSeqTM 4000 platform (Gene Denovo, Guangzhou, China).

### Bioinformatics Analysis

The differentially expressed mRNAs between the two groups were screened using the edgeR software with FDR (false discovery rate) < 0.01 and log2FC (fold change) >1 as the thresholds for significant differences. The DEmRNAs were functionally annotated by gene ontology (GO) using the R software ClusterProfile package, and KEGG pathways using the Metascape software (http://metascape.org/gp/index.html#/main/step1) (15).

### Construction of the lncRNA-miRNA-mRNA Network

The DElncRNAs and DEmRNAs between the non-tumor and SEM, non-tumor and non-SEM, and non-tumor and non-SEM+SEM groups were identified using the edgeR software as described above. The common DElncRNAs and DEmRNAs among the three comparison groups were screened, and those with Spearman correlation coefficient r2>0.9999 and p<0.001 were selected for network construction. The target miRNAs of the lncRNA-mRNA interaction pairs were predicted using the R software miRNAtap package, TargetScan (16) and Miranda (17). The miRNAs obtained from all three programs were used to construct the lncRNA-miRNA-mRNA network, which was visualized using Cytoscape_v3.8.0.

### Public Database Data Mining

The DEmRNAs were validated using the GEPIA2 database (http://gepia2.cancer-pku.cn/#index) (18). The RFS of the different groups (total cases 134) were plotted using the Kaplan-Meier Plotter (http://kmplot.com/analysis/index.php?p=background), and compared in terms of P values and hazard ratios (HR) with 95% confidence intervals (CI) by the Log-rank test and univariate Cox proportional hazard regression (19).

### Risk Factor Analysis

RNA-sequencing data (level 3) of 134 tumors and corresponding clinical information were obtained from The Cancer Genome Atlas (TCGA) dataset (https://www.cancer.gov/). The predictive accuracy and risk score of genes were compared by TimeRoc analysis. The minimum absolute shrinkage and selection operator (Lasso) regression algorithm was used for feature selection with 10-fold cross-validation using the R software package GLMnet. All statistical analyses were performed using R software (R Foundation for Statistical Computing, 2020) version V4.0.3. P <0.05 was considered statistically significant.

### Gene Set Variation Analysis

The genomic gene set analysis in TGCT was used the Gene Set Cancer Analysis (GSCA) (http://bioinfo.life.hust.edu.cn/GSCA/#/), which is an integrated database for genomic and immunogenomic gene set cancer analysis (20). Immunogenomic analysis was performed by ImmuCellAI (Immune Cell Abundance Identifier) algorithm with 24 immunes cells (http://bioinfo.life.hust.edu.cn/ImmuCellAI#!/) (21). P <0.05 was considered statistically significant.

## Results

### Identification of Differentially Expressed mRNAs in TGCT

**Figure 1:**
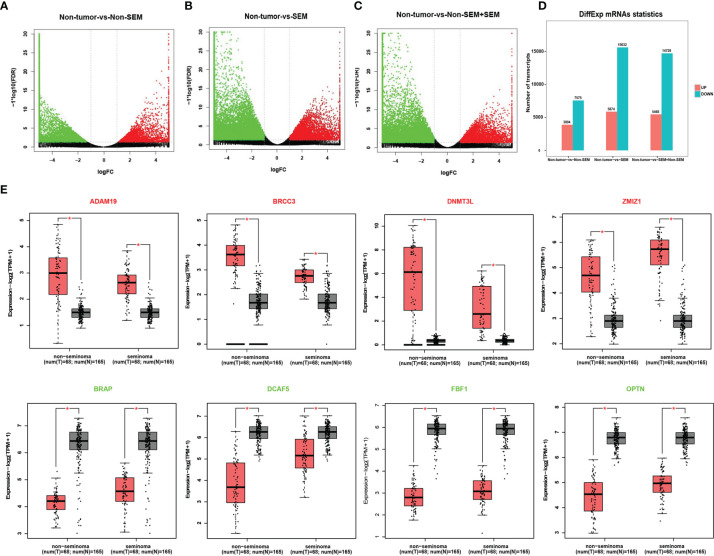
Identification of DEmRNAs across different groups. **(A-C)**. Volcano plots showing the DEmRNAs between **(A)** non-tumor *vs* non-SEM, **(B)** non-tumor *vs* SEM, and **(C)** non-tumor *vs* SEM and non-SEM with |Log2FC|>1 and FDR <0.01. **(D)** Histogram showing the DEmRNAs between groups. **(E)** The validation of randomly selected DEmRNAs in the GEPIA database with |Log2FC|>1 and p <0.01 as the cut-off values. *Difference was statistically significant.

A total of 11,469 mRNAs were differentially expressed between the non-tumor and non-SEM samples, of which 3,894 were up-regulated and 7,575 were down-regulated in the latter (**Figures 1A, D**). Furthermore, there were 21,506 DEmRNAs in SEM relative to the non-tumor samples, of which 5974 were up-regulated and 15,632 were down-regulated (**Figures 1B, D**). Finally, the SEM and non-SEM tumors exhibited 20,197 DEmRNAs, including 5,468 up-regulated and 14,729 down-regulated mRNAs compared to the non-tumor samples (**Figures 1C, D**). The expression levels of the randomly selected ADAM19, DNMT3L, BRCC3, ZMIZ1, BRAP, DCAF5, FBF1 and OPTN were verified using the GEPIA2 database (**Figure 1E**).

### Expression Pattern of Top 100 mRNAs in TGCT

**Figure 2:**
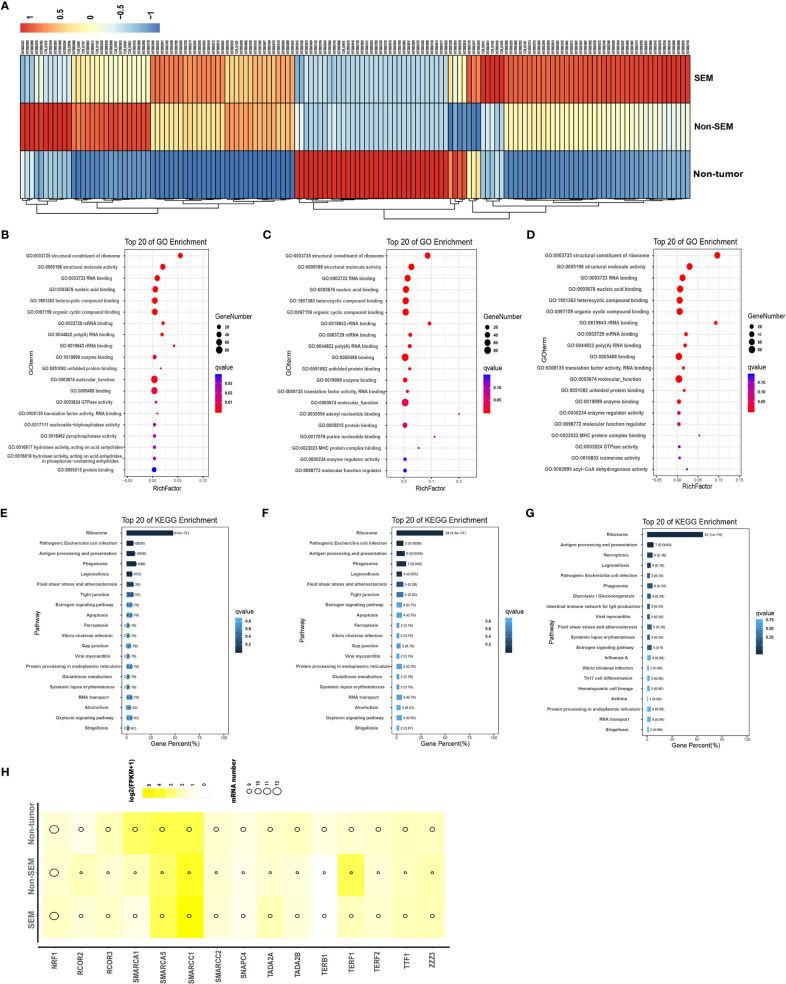
Intergroup-specific expression and functional enrichment analysis of mRNAs. **(A)** The heatmap of top 100 highly expressed mRNAs in the non-tumor, SEM and non-SEM samples. **(B–G)**. The top 20 GO terms and KEGG pathways in non-tumor **(B, E)**, SEM **(C, F)**, and non-SEM **(D, G)** groups. **(H)** Bubble chart of putative transcription factors of the top 100 mRNAs in three groups.

To determine the contribution of DEmRNAs to TGCT, we analyzed the top 100 mRNAs with high expression levels in the non-tumor (vs SEM + non-SEM), SEM (vs non-tumor + non-SEM), and non-SEM (vs non-tumor + SEM) samples (**Figure 2A**). The highly expressed genes in the non-tumor samples were enriched in the GO terms such as “structural constituent of ribosome”, “structural molecule activity” and “nucleoside-triphosphatase activity” (**Figure 2B**), and the “Tight junction”, “Gap junction” and “Apoptosis” pathways (**Figure 2E**). In the non-SEM samples, the significantly enriched GO terms were “molecular function”, “MHC protein complex binding” and “translation factor activity, RNA binding” (**Figure 2C**), whereas the main KEGG pathways were enriched in immune-related signaling pathways, such as “B cell receptor signaling pathway”, “Antigen processing and presentation” and “intestinal immune network for IgA production” (**Figure 2F**). The highly expressed genes in SEM showed significant enrichment in “MHC protein complex binding” “structural constituent of ribosome” and “molecular function” (**Figure 2D**), and the immune-related signaling pathways, such as “Antigen processing and presentation”, “intestinal immune network for IgA production” and “Th17 cell differentiation” (**Figure 2G**). Furthermore, the top 100 highly expressed mRNAs in all groups were associated with multiple transcription factors, including SMARCA5 and SMARCC1, which were overexpressed in all three groups (**Figure 2H**).

### Enrichment Analysis of Common DEmRNAs

**Figure 3:**
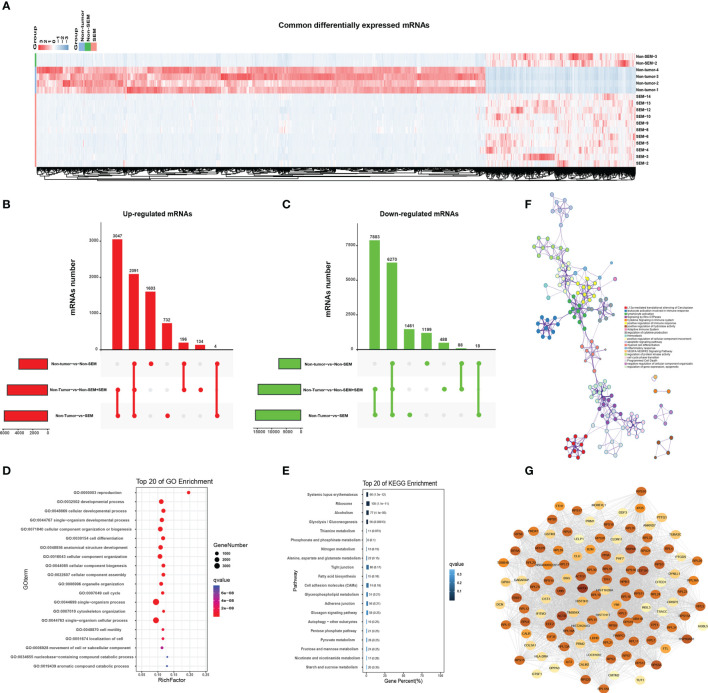
Enrichment analysis of common DEmRNAs. **(A)** Heat map showing hierarchical clustering of common DEmRNAs in the non-tumor, SEM and non-SEM groups. **(B, C)** Upset plots showing the distribution of **(B)** upregulated and **(C)** downregulated mRNAs for the indicated pairs. **(D, E)** Bar graph showing the top 20 enriched GO and KEGG pathways associated with DEmRNAs common to all three pairs. **(F)** The interactive network of the enriched pathways. Different colors represent different biological processes. **(G)** The protein-protein interaction network (PPI) of the top 100 common DEmRNAs. Color intensity is indicative of the number of connections.

As shown in **Figure 3A**, there were many down-regulated mRNAs in SEM and non-SEM samples compared to the controls (**Figure 3A**). Specifically, 3047 mRNAs were up-regulated and 7,883 mRNAs were downregulated in the SEM and total TGCT (SEM + non-SEM) samples relative to the non-tumor controls (**Figures 3B, C**). The non-SEM and combined TGCT samples showed upregulation of 196 mRNAs and downregulation of 88 mRNAs compared to the non-tumor group. Four upregulated and 18 downregulated mRNAs were common to both SEM and non-SEM groups compared to the non-tumor control (**Figures 3B, C**). A pairwise comparison of all groups revealed that 2,091 upregulated mRNAs and 6,270 downregulated mRNAs were common. In addition, 1,603 and 1,191 mRNAs were respectively upregulated and downregulated only in the non-SEM group relative to the non-tumor group, whereas 732 upregulated and 1,461 downregulated mRNAs were specific to the non-tumor-vs-SEM comparison, and 134 upregulated and 488 downregulated mRNAs were only detected when comparing non-tumor samples to all TGCT samples (**Figures 3B, C**). GO analysis revealed that the DEmRNAs common to all three groups were enriched in “cell motility”, “movement of cell or subcellular component” and “cell cycle” (**Figure 3D**), and the top 20 pathways were “cell adhesion molecules”, “adherence junction” and “Tight junction” (**Figure 3E**). Furthermore, the signaling pathways significantly associated with the top 100 common DEmRNAs included “apoptosis signaling pathway”, “positive regulation of cellular component movement”, “leukocyte activation involved in immune” and “cytokine signaling in immune system”, and were interconnected (**Figure 3F**). The protein-protein interaction (PPI) network of the top 100 common DEmRNAs revealed 3,098 connections (**Figure 3G**).

### Construction and Analysis of the lncRNA-miRNA-mRNA Network

**Figure 4:**
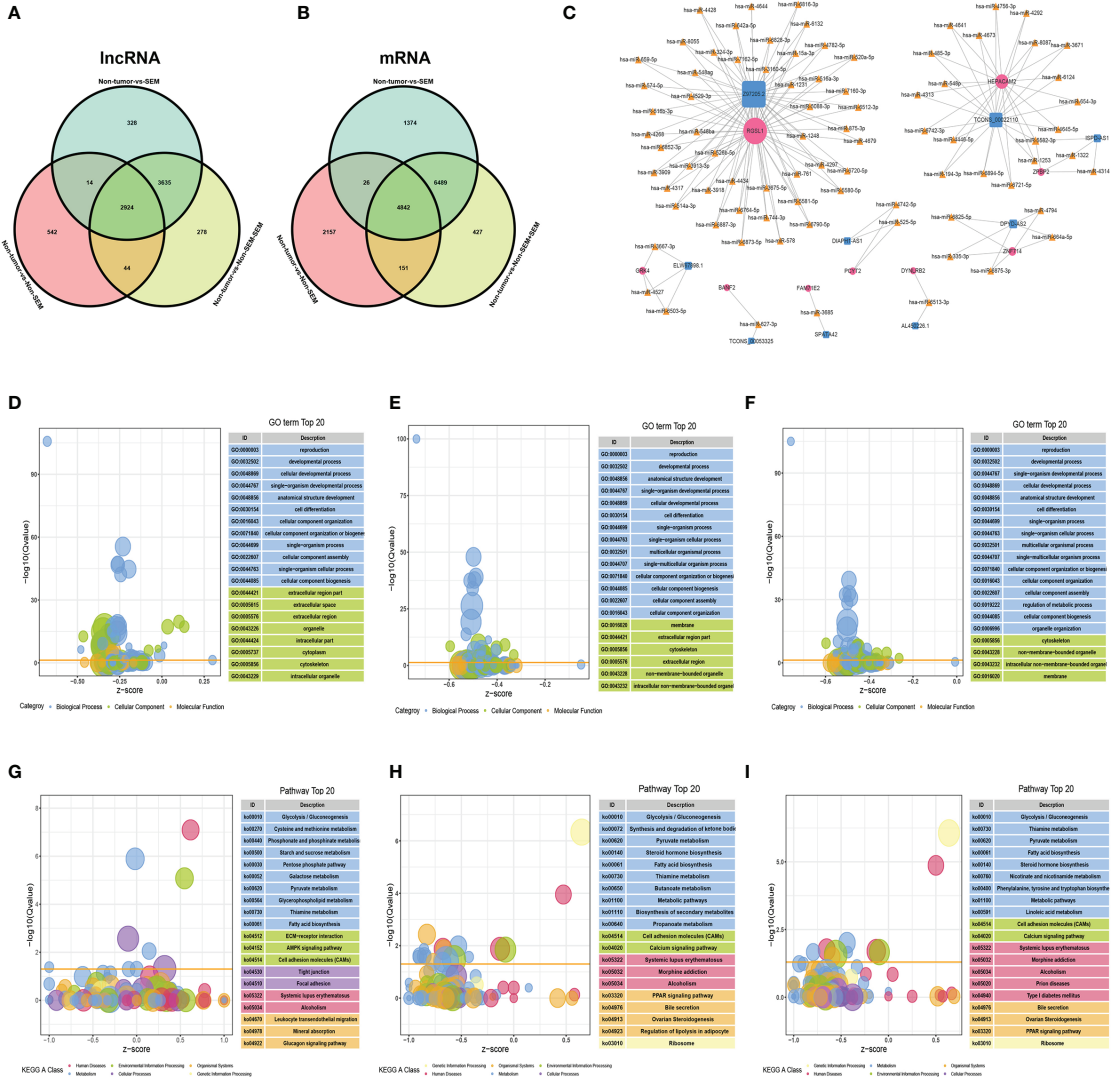
The lncRNA-miRNA-mRNA ceRNA network in TGCT. **(A, B)** Wayne diagrams of DElncRNAs **(A)** and DEmRNAs **(B)** in three comparison groups, with |log2FC|>2, FDR<0.01 as the thresholds. **(C)** The lncRNA–miRNA–mRNA network consisting of 9 lncRNAs (blue squares), 82 miRNAs (yellow triangles) and 9 mRNAs (rose-red circles) with 166 possible interactions. **(D–I)** Enrichment analysis of the DEmRNAs in **(D, G)** non-tumor *vs* non-SEM, **(E, H)** non-tumor *vs* SEM, and **(F, I)** non-tumor *vs* SEM + non-SEM. The top 20 enriched GO terms and pathways are shown.

A total of 2,924 DElncRNAs and 4,842 DEmRNAs were common to the non-tumor vs SEM, non-tumor vs non-SEM+SEM, and non-tumor vs non-SEM comparisons (**Figures 4A, B**). The correlated mRNA-lncRNA pairs were screened with R2 > 0.9999 and p < 0.001 as the criteria. Nine lncRNAs were identified, including SPATA42, AL450226.1, TCONS_00053325, DPYD-AS2, ELW67898.1, DIAPH1-AS1, Z97205.2, ISPD-AS1 and TCONS_00022110. The nine mRNAs were FAM71E2, HEPACAM2, RGSL1, DPYD-AS2, ZPBP2, DYNLRB2, PCYT2, BANF2, and GRK4. The regulatory miRNAs of the candidate mRNAs and lncRNAs were predicted by the R software miRNAtap package, TargetScan (16), and Miranda (17). We identified 8 lncRNA–miRNA–mRNA regulatory networks that included 9 lncRNAs nodes, 9 mRNAs nodes, and 82 miRNAs nodes with 166 possible interactions (**Figure 4C**). The DEmRNAs common to non-tumor vs non-SEM and non-tumor vs SEM pairs were associated with “movement of cell or subcellular component”, “reproduction” and “cell motility” both in the groups of (**Figures 4D, E**), whereas “male gamete generation”, “cell differentiation” and “reproduction progress” were the functions enriched in the DEmRNAs between non-tumor vs non-SEM + SEM (**Figure 4F**). The top 20 enriched pathways among the DEmRNAs between non-tumor vs non-SEM groups were “focal adhesion” and “cell adhesion molecules” (**Figure 4G**), that for non-tumor vs SEM were “biosynthesis of secondary metabolism”, “steroid hormone biosynthesis” and “cell adhesion molecules” (**Figure 4H**), and “cell adhesion molecules” and “metabolic pathway” were enriched for non-tumor *vs* non-SEM + SEM (**Figure 4I**).

### Evaluation of Clinical Outcomes

**Figure 5:**
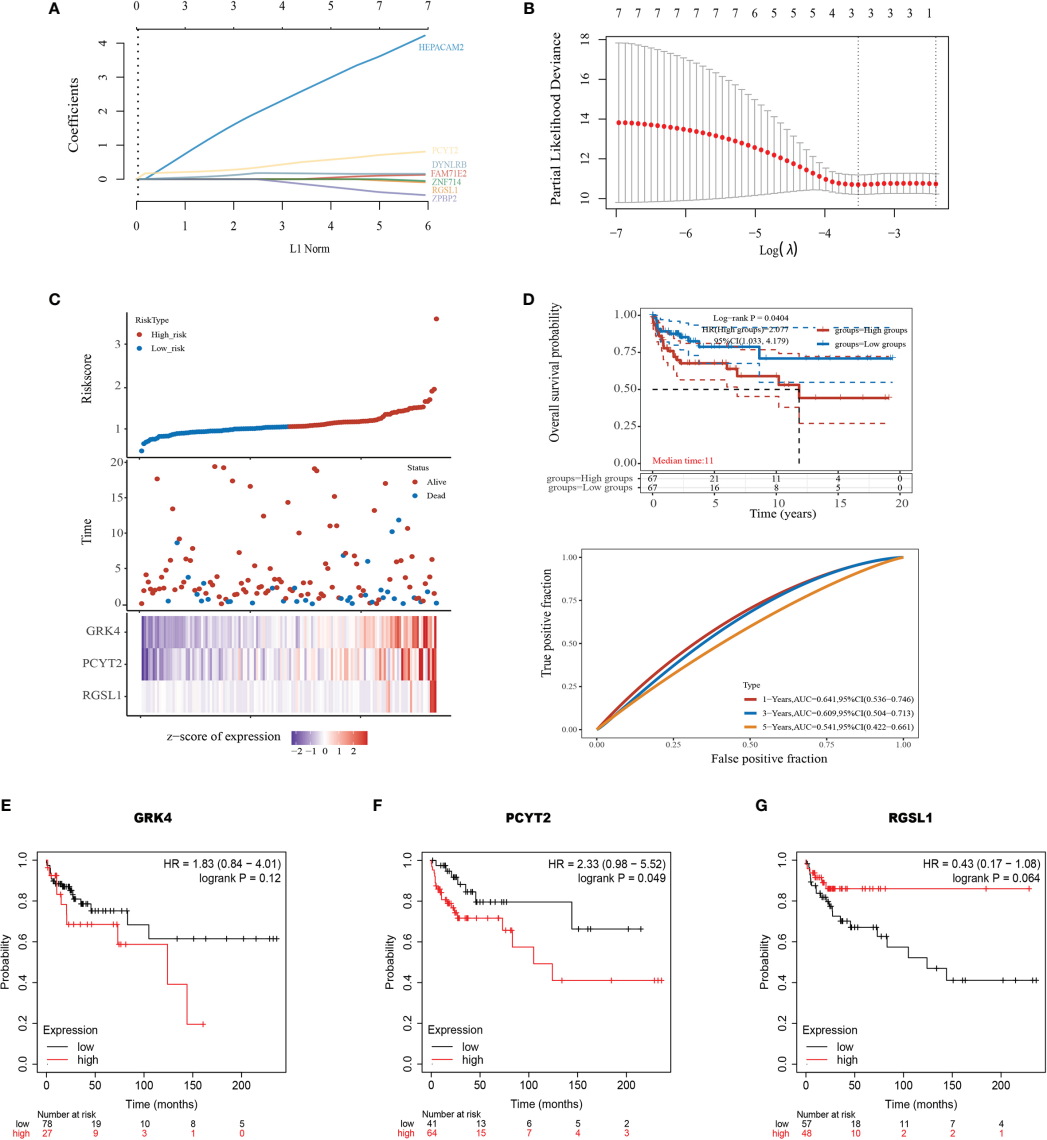
Development of the prognostic index based on ceRNA-related genes. **(A)** The coefficient of the selected feature is shown by the lambda parameter, with the value of the independent variable lambda on the horizontal axis and the coefficient of the independent variable on the vertical axis. **(B)** The relationship between partial likelihood deviation and log (λ) was plotted using the Lasso Cox regression model. **(C)** Risk score curve showing survival of patients and expression profiles of the three prognostic genes in low- and high-risk groups. **(D)** Kaplan-Meier curve showing the association between patient survival and the three-gene signature. Median survival duration (years) corresponds to a 50% survival rate. The ROC curve and AUC of the risk model at different times. **(E–G)** Survival of patients stratified based on GRK4, PCYT2, and RGSL1 expression.

To explore the clinical value of the nine mRNAs (see above) in TGCTs, we obtained the raw RNA-Seq data (Level 3) of 134 TGCTs and the corresponding clinical information from TCGA dataset (https://www.cancer.gov/) (**Supplementary Table 1**). A polygenic risk score (PRS) was then calculated based on the correlation between the expression levels of the nine mRNAs and prognosis using the LASSO cox regression analysis (**Figures 5A, B**). Accordingly, the patients were divided into the high-risk and low-risk groups (**Figure 5C**, top). Overexpression of GRK4, PCYT2 and RGSL1 was associated with increased mortality rates (**Figure 5C**, middle) and a higher risk score (**Figure 5C**, bottom). The prognostic index was calculated as follows:


Risk score=(1.841)*RGSL1+(0.1582)*PCYT2+(0.3161)*GRK4

KM survival analysis also confirmed that the high expression level of this gene set portended poor prognosis (log-rank p=0.0404, HR=2.077, **Figure 5D**). Furthermore, the area under the time-dependent ROC curve of the gene set was greater than 0.5, indicating good predictive power (**Figure 5D**). To further establish the prognostic value of GRK4, PCYT2 and RGSL1, we analyzed the correlation between their expression levels with RFS using Kaplan-Meier Plotter. PCTY2 (P=0.049, **Figure 5F**) expression level showed a significant negative correlation with RFS, while that of GRK4 (P=0.12, **Figure 5E**) and RGSL1 (P=0.064) were negatively correlated with RFS without statistical significance (**Figures 5F, G**).

### GSCA of GRK4, PCYT2, and RGSL1

**Figure 6:**
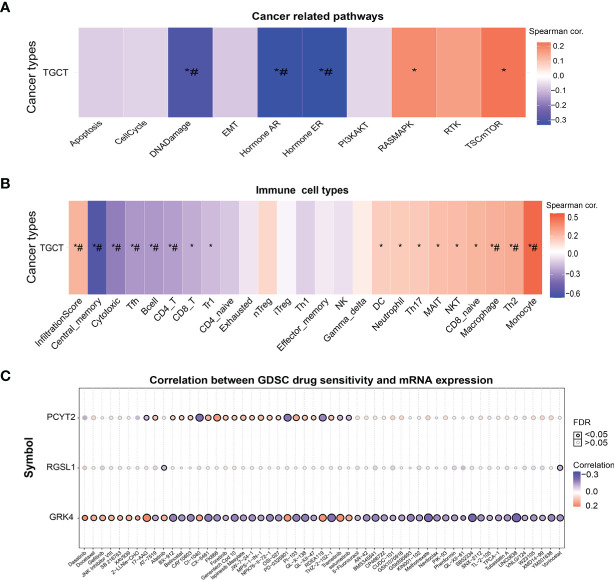
The correlation of GRK4, PCYT2 and RGSL1 with cancer-related pathways, immune cell infiltration and GDSC. **(A)** Association between GSVA score and cancer-related pathways in TGCT. **(B)** Correlation between immune cell infiltrates and GSVA enrichment score in the selected cancers. **(C)** Correlation between gene expression and GDSC drug sensitivity. *P-value < 0.05; ^#^FDR < 0.05.

The potential therapeutic relevance of GRK4, PCYT2 and RGSL1 was evaluated by GSCA (http://bioinfo.life.hust.edu.cn/GSCA/#/) (20) and Spearman correlation analysis. As shown in **Figure 6A**, the expression levels of this gene set in TGCT showed a significant negative correlation with DNA damage, androgen receptor (AR), estrogen receptor (ER) and other pathways, and positive correlation with RASMAPK, TSCmTOR and other pathways (**Figure 6A**). Furthermore, the gene set was also negatively correlated with the infiltration of immune cells such as Cytotoxic, TFH, B cell, CD4_T and CD8_T, and positively with Monocyte, Th2 and Macrophage (**Figure 6B**, **Supplementary Tables 2, 3**).

Finally, we analyzed the correlation between drug sensitivity of cancer cell lines in GDSC (Genomics of Drug Sensitivity in Cancer), and the GRK4, PCYT2 and RGSL1 expression levels (22). GRK4 was correlated with the target drug 17-AAG and increased sensitivity to RDEA119, PD-0325901, CX5461, Trametinib and Selumetinib. PCYT2 showed a significant positive correlation with sensitivity to targeted drugs such as CX-5461, PI-103 (PI3K and mTOR inhibitors) and MPS-1-IN-1 (**Figure 6C**).

## Discussion

Although previous studies have identified the hereditary nature of TGCT with an estimated 37–49% familial cases (23, 24), the only moderate penetrance gene is GHEK2 whose pathogenic variants have been associated with the risk of TGCT (25). The testis expresses the largest number of genes of any mammalian organ (26), especially high numbers of genes predominantly expressed (27). In our study, it also had numerous DEmRNAs in TGCTs. We identified several dysregulated mRNAs in TGCT tissues, which are likely involved in tumor development and progression. The upregulated mRNAs were functionally annotated to immunomodulation, cell migration and invasion, indicating a correlation between infiltration of immune cells and TGCT metastasis. The dynamics of the immune microenvironment in the scoring model based on stroma and immune cell infiltration are essential factors predicting cancer prognosis and chemotherapy response (28). Tumor-infiltrating immune cells are the primary immune signatures closely related to the clinical outcomes of immunotherapies (29). Therefore, we explored the influence of immune cells on the efficacy of immunotherapy and the clinical progression of patients with TGCT. GRK4, PCYT2 and RGSL1 were negatively correlated with the infiltration of Cytotoxic, TFH, B cell, CD4_T and CD8_T populations, and positively with that of Monocyte, Th2 and Macrophage. Recently, a study also reported that advanced TGCT is associated with a decrease in T cells and NK cells, and increased infiltration of Tregs, neutrophils, mast cells and macrophages (30). Furthermore, chimeric antigen receptor T (CAR-T) cells also showed anti-tumor activity against metastatic EC xenografts in a mouse model (31). Our results showed that upregulated mRNAs in TGCTs are likely involved in immunomodulation and immune cell infiltration, and thus promising therapeutic targets.

Numerous studies have shown that lncRNAs can compete with other RNAs to bind miRNAs, and function as ceRNAs to regulate the expression of target mRNAs (32). CeRNAs are repeatedly dysregulated in cancer, and involved in tumor initiation and progression (14). We constructed a lncRNA-miRNA-mRNA ceRNA regulatory network with the TGCT DEmRNAs and DElncRNAs and identified HEPACAM1 (TCONS_00185248) as one of the hub genes. As other studies have shown, hepatocyte cell adhesion molecules 1 and 2 (HEPACAM1 and 2) are members of the immunoglobulin family, and inhibit cell cycle progression in breast cancer via p53, p21 and p27 signaling (33). In addition, the nine mRNAs of the ceRNA regulatory networks included GRK4, PCYT2 and RGSL1, which showed prognostic relevance and was associated with cancer-related pathways such as DNA Damage, AR, ER, RASMAPK, and TSC-mTOR. A meta-analysis also showed that a relative reduction in androgens compared to the general population increases the risk of TGCT (34). Other studies have reported that the TSC1/2-mTOR pathway regulates the function of diverse immune cells, along with cell growth and metabolism (35–37). In our study, the expression level of the gene set was correlated with the infiltration of various immune cells and therefore may regulate the immune responses to TGCT via the TSC1/2-mTOR pathway. Furthermore, GRK4 and PCYT2 were correlated with the sensitivity to multiple chemotherapeutic drugs, and PCYT2 showed a significant negative correlation with RFS in TGCT patients. Thus, GRK4, PCYT2 and RGSL1 are reliable prognostic markers and potential therapeutic targets for TGCTs.

We constructed a ceRNA network for TGCT based on the DEmRNAs and DElncRNAs for the first time, and identified markers involved in TGCT prognosis and chemotherapy resistance, which can help select the optimum clinical regimen. However, there are some limitations in our study that ought to be considered. First, our conclusions are only based on bioinformatics analysis and other methods, and further experimental verification is needed. Second, our sequencing data lacks the expression information of miRNAs, since the sample is not enough to detect the level of miRNAs. Third, patients were enrolled from only one hospital, the possible influence of regional and ethnic factors cannot be eliminated. In addition, the sample size in this study was relatively small. Finally, the involvement of GRK4, PCYT2 and RGSL1 in cancer-related pathways and drug susceptibility needs further experimental validation. In summary, our findings provide new insights into the pathogenesis of TGCT and identify GRK4, PCYT2 and RGSL1 as key prognostic markers and therapeutic targets.

## Conclusion

We identified several dysregulated mRNAs in TGCT that are related to immunomodulation, cell migration and invasion. In addition, GRK4, PCYT2 and RGSL1A are potential TGCT-specific biomarkers that can predict RFS, tumor immunity and chemotherapeutic resistance.

## Data Availability Statement

Publicly available datasets were analyzed in this study. This data can be found here: https://www.cancer.gov/about-nci/organization/ccg/research/structural-genomics/tcga.

## Ethics Statement

The studies involving human participants were reviewed and approved by Hunan Cancer Hospital, Department of Urology, The Affiliated Cancer Hospital of Xiangya School of Medicine of Central South University. The patients/participants provided their written informed consent to participate in this study.

## Author Contributions

HB, LT, and LF conceived and planned the experiments. FZ, DZ, and ZL collected clinical samples. HB, FZ, and ZL analyzed and interpreted the data. FZ and HB drafted the manuscript. HB, JF, and LT proofed the manuscript. LF revised and confirmed the final manuscript. All authors contributed to the article and approved the submitted version.

## Funding

This research was funded by the National Key Research and Development Program of China (Nos. 2016YFC1000600), Changsha Municipal Natural Science Foundation (Nos. kq2014033), Hunan Provincial Grant for Innovative Province Construction (Nos. 2019SK4012), and the Fundamental Research Funds for the Central Universities of Central South University (Nos. 160171016).

## Conflict of Interest

Authors DZ, LX, HB and LF were employed by the company China International Trust and Investment Corporation (CITIC).

The remaining authors declare that the research was conducted in the absence of any commercial or financial relationships that could be construed as a potential conflict of interest.

## Publisher’s Note

All claims expressed in this article are solely those of the authors and do not necessarily represent those of their affiliated organizations, or those of the publisher, the editors and the reviewers. Any product that may be evaluated in this article, or claim that may be made by its manufacturer, is not guaranteed or endorsed by the publisher.
